# Giant cell tumor of tendon sheath in the wrist that damaged the extensor indicis proprius tendon: a case report and literature review

**DOI:** 10.1186/s12885-019-6293-x

**Published:** 2019-11-06

**Authors:** Qingfang Zhao, Hui Lu

**Affiliations:** 1 Department of Plastic Surgery, The First Affiliated Hospital, Zhejiang University, #79 Qingchun Road, Hangzhou, Zhejiang Province 310003 People’s Republic of China; 2 Department of Orthopedics, The First Affiliated Hospital, Zhejiang University, #79 Qingchun Road, Hangzhou, Zhejiang Province 310003 People’s Republic of China

**Keywords:** Giant cell tumor of tendon sheath(GCTTS), Impairment of tendon, Extensor indicis proprius tendon(EIP tendon)

## Abstract

**Background:**

Giant cell tumor of the tendon sheath (GCTTS) is a benign soft tissue (synovial membrane) tumor that rarely involves the hands or wrists. And Tendon impairment caused by GCTTS is extremely rare.

**Case presentation:**

Here, we reported a case of a 60-year-old female with a 10-year history of gradually increasing mass in her left dorsal wrist. The EIP tendon was partially impaired by the mass.The patient was treated with surgical excision of the mass and reconstruction of the EIP tendon. The histopathological examination suggested the presence of GCTTS. After surgery, the patient had adequate functional recovery and no tumor recurrence after 2 years’ follow-up.

**Conclusion:**

GCTTS in hands and wrists rarely damages the tendon. Early diagnosis and proactive interventions may likely contribute to good prognostic outcomes.

## Background

Giant cell tumor of the tendon sheath (GCTTS), which originally arises from the synovial cells of tendon sheaths or tendinous spaces, is a slow progressing benign tumor [1–3]. GCTTS can be categorized into two types based on morphology [4, 5]—located nodular type (usually located in the digits and wrists and surrounded by a pseudocapsule) and diffuse type (commonly found around large joints). The located nodular type is more prevalent and patients with the located nodular type generally have a long history of tumors that frequently occur as peritendinous fibrous masses in the digits and wrists [6]. In contrast, the diffuse-type GCTTS, formerly known as Pigmented Villonodular Synovitis (PVNS) [7], is more aggressive and mainly involves the larger joints such as the knees [8], hips [9], ankles [10], and elbows [11]. The located nodular type is usually benign, while, the diffuse type is more invasive, and may occasionally become malignant. However, very few reports [1, 5, 7, 12] have indicated tendon damage due to GCTTS. A damaged tendon can cause serious harm and even result in disabilities. Here, we present a case report of localized GCTTS in the wrist. This has rarely been reported before. Since there was a lack of similar documented cases, the damage to the patient’s extensor indicis proprius tendon could not be prevented.

## Case presentation

**Fig. 1:**
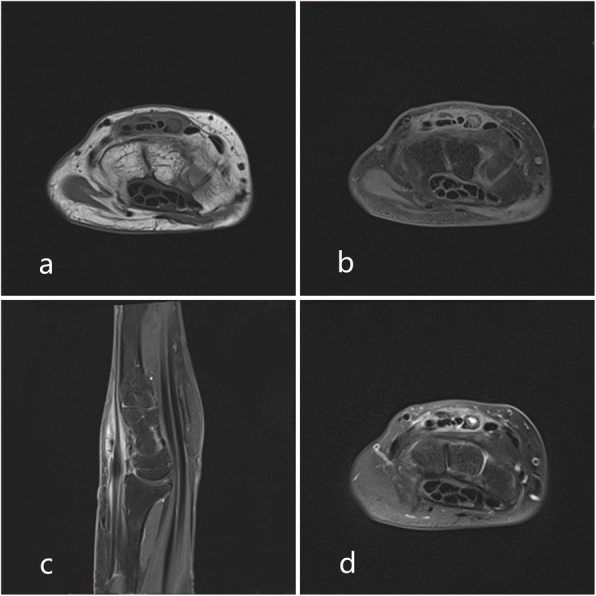
MRI (**a,b,c**) reveals a 18 × 6× 5 mm spindle-like mass,encircles the extensor indicis proprius tendon in her left dorsal wrist.The lesion shows iso-indensity on T1 image (1a),while it shows hyperintense signal on T1WI with fat suppression(**b**: axial plane; **c**: sagittal plane) and T2WI with fat suppression (**d**).On T2WI with fat suppression (**d**), we can see the mass (shows hyperintense signal in the center and iso-indensity in the peripheral) attached to the EIP tendon(shows hypointense signal)

**Fig. 2:**
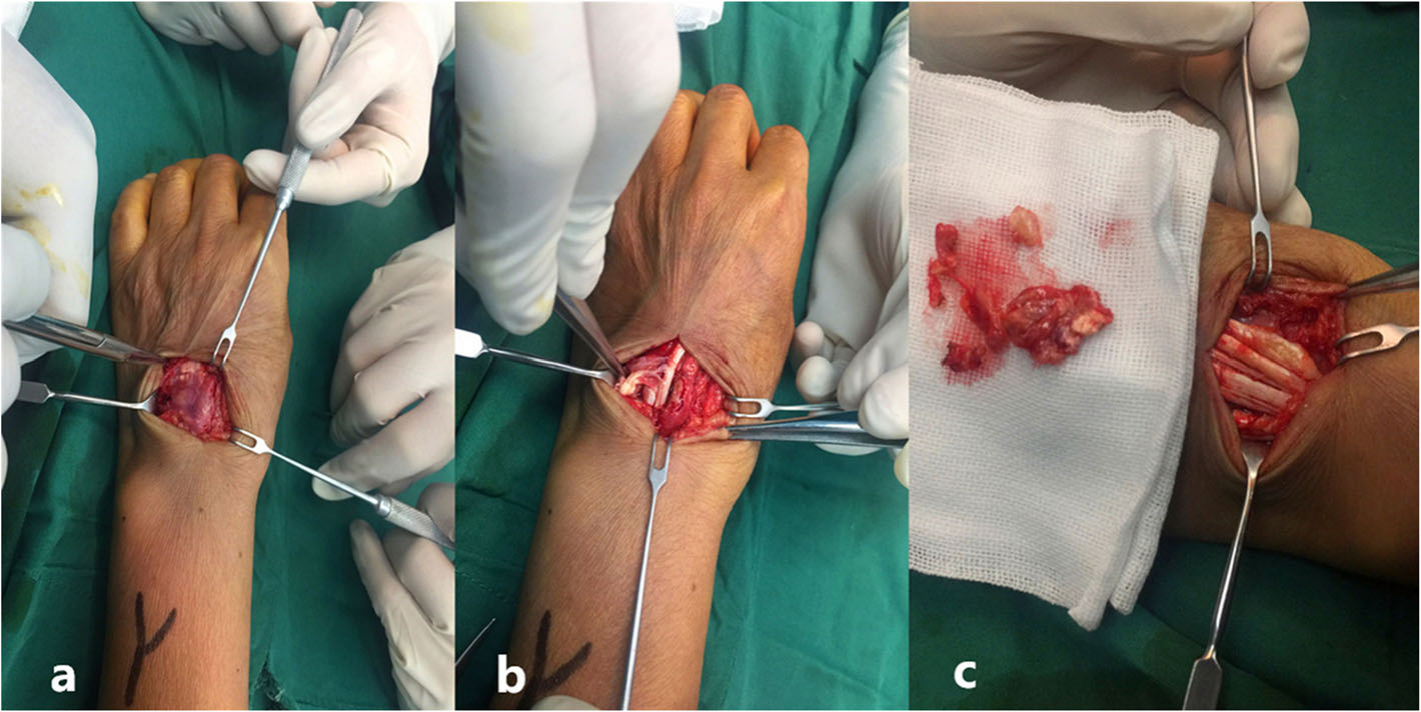
Intraoperative photograph (**a**) shows that the lesion was at the surface of the extensor indicis proprius tendon and that the tendon was partly infiltrated; (**b** and **c**): even though the tumor had a capsule, it partly infiltrated and destroyed the tendon

**Fig. 3:**
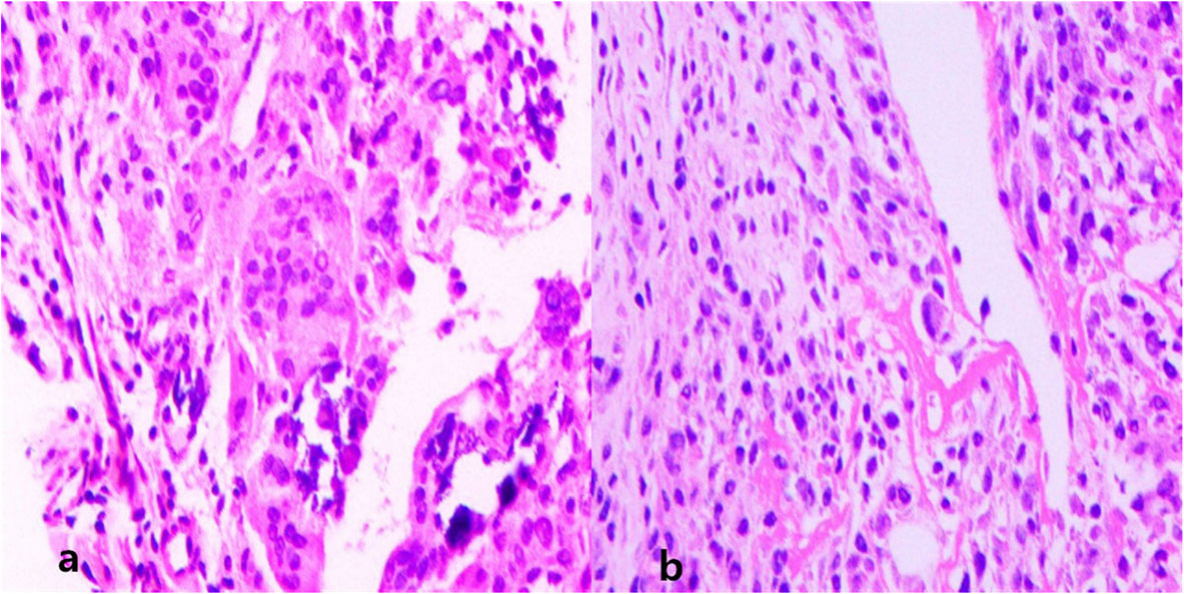
Pathological examination of the tumor using hematoxylin–eosin stain. **a** Multinucleated giant cells were distributed in the interstitial tissue; (**b**) the synovial mononuclear cells were pervaded

**Fig. 4:**
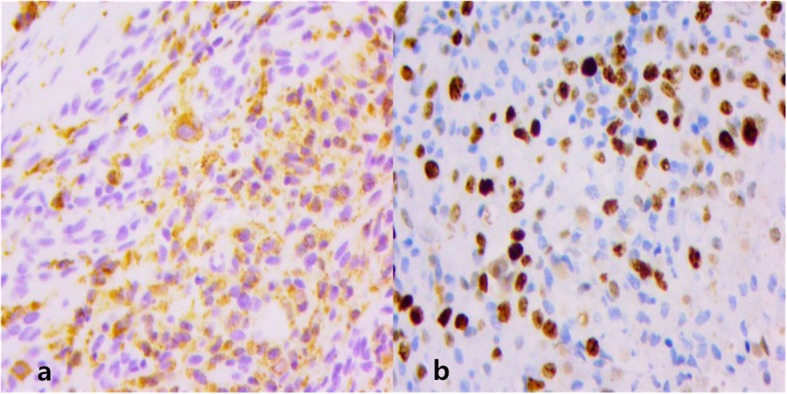
Pathological examination of tumor using immunohistochemistry staining. **a** Monocytes with positive CD68. **b** Monocytes with positive Ki-67

We report a 60-year-old female patient presented with an asymptomatic nodular lesion in her left dorsal wrist over the 10 years. The mass had grown gradually from the size of a green pea initially, and no history of trauma was mentioned. The mass slightly weakened the degree of dorsal extension of her left wrist, but did not cause any pain or numbness. Upon local examination, we found that the mass was firm, non-tender, and located on the dorsal side of the left wrist. It measured 5 × 6 × 18 mm. The mass was free from the skin but was attached to the extensor indicis proprius tendon. The range of motion of her wrist was 45° extension to 80° flexion. The results of the laboratory studies were as follows: the blood routine, antistreptolysin O (ASO) level, erythrocyte sedimentation rate (ESR), high-sensitivity C-reactive protein (hs-CRP) level, anti-cyclic peptide containing citrulline (anti-CCP) level, and other immune indices were normal. The levels of biological markers of the tumor were also normal. MRI scans (Fig. 1) showed a mass in her left dorsal wrist, arising from the extensor indicis proprius tendon, and a partial tear in the left wrist triangular fibrocartilage disc. The lesion shows iso-indensity on T1 image (1a),while it shows hyperintense signal on T1WI with fat suppression(1b,1c) and T2WI with fat suppression(1d).On T2WI with fat suppression(1d), we can see the mass (shows hyperintense signal in the center and iso-indensity in the peripheral) attached to the EIP tendon(shows hypointense signal).The MRI scans were helpful in planning an appropriate surgical approach. The location and extent of involvement of the tumor was further evaluated using MRI. The involvement of the adjacent soft tissues, joints, and tendon sheaths were also evaluated. We recommended a biopsy in order to confirm our diagnosis before proceeding with the treatment. Unfortunately, the patient declined the biopsy. We performed the surgery under brachial plexus block anesthesia. During the surgery (Fig. 2), we found that the mass was located exclusively on the surface of the left extensor indicis proprius tendon. Even though the tumor had a capsule, it had partially infiltrated and damaged the tendon. The tendon sheath was impaired. We carefully removed the infiltrated part of the EIP tendon and checked whether the residual tendon could be treated by suturing. Fortunately,the residual EIP tendon could be repaired by direct suturing. Analysis of the frozen sections revealed ganglions associated with synovial cell proliferation, while the histological findings (Fig. 3 and Fig. 4) revealed GCTTS,and partially invading the EIP tendon. In addition, the micrograph showed numerous short shuttle-like tumor cells, with sporadic multinuclear giant cells and multiple calcifications in the matrix, and the immumohistochemical staining presented CD68(+), Ki-67(+), S-100(focal+), CK(pan)(−),SMA(−). The patient underwent radical resection of the tumor without radiotherapy and demonstrated adequate functional recovery.Above all,no recurrence was found after 2 years’ follow-up.

## Conclusion and discussion

The etiology of GCTTS has not yet been fully elucidated. Some possible etiologies include disturbances in lipid metabolism, neoplasms, inflammation, trauma, and hemorrhage [13, 14]. However, only two of these etiologies (inflammatory and tumoral) are commonly discussed [15]. Pathologically, GCTTS is composed of synovial mononuclear cells and osteoclast-like multinucleated giant cells [12]. Some physicians and researchers believe that the presence of increased cellularity and the tendency to recur imply neoplastic origin [16, 17]. There are several studies that discuss the importance of genetic factors in the occurrence of GCTTS. De Jong B et al. proved that the existence of trisomy 7 [18] is associated with GCTTS, suggesting the neoplastic aspect in the proliferation [19] of GCTTS (trisomy 7 is associated with a large variety of neoplastic conditions [20]). Similarly, infiltration by histiocytes, macrophages, and plasmatic cells support the possibility of an inflammatory origin [13]. The increased activity of the epidermal growth factor (EGFR) and the growth factor, PDGF, codified by c-erb B oncogenes on chromosome 7, may also contribute to the inflammation caused by synovitis [18–20]. We found very few reports [7, 11, 12] of the destruction of tendon as a result of GCTTS. Localized GCTTS commonly occurs in the form of a well-delineated lesion that encircles the tendon instead of infiltrating it. On the contrary, diffuse GCTTS is usually aggressive, occurs as a homogeneous soft-tissue mass and is associated with joint destruction and deterioration [14]. In the limbs, the lesion is contained within anatomically narrow spaces, and can grow along the sheaths causing synovium degeneration and cortical erosion. In the case reported here, the destruction of the extensor indicis proprius tendon was irregular and lacked a well-defined etiology.

While GCTTS is still considered a rare benign clinical disease, it has become more common in recent times. GCTTS can be classified as either localized or diffuse based on its clinical presentation and biological behavior; the latter is known to be more aggressive. In accordance with previously published literature, we diagnosed this case of GCTTS in the wrist as a benign and noninvasive tumor. However, during surgery, we found that the lesion had surrounded, partly infiltrated, and damaged the extensor indicis proprius tendon. Such observations are extremely rare. In this case, the tendon injury seen could not have been iatrogenic because the patient had not received any prior treatments (e.g. surgery, corticosteroid injection). In addition, what’s the possible reasons resulting in the repture of the EIP tendon? We thought that the likely cause of the EIP tendon rupture was combination of GCTTS infiltrating into the tendon and also the mechanical or pressure effect of the EIP tendon excursion under the wrist extensor retinaculum.So decreasing the movement of the left wrist may be helpful to protect the EIP tendon in this case.Intraoperative view, we found that the strength of extensor indicis proprius tendon was satisfactory.We transected part of the affected tendon and sutured longitudinally. Three days after surgery, the patient began functional training and rehabilitation under the guidance of a doctor. The patient recovered well, had no functional impairments, no tendon re-rupture or adhesion, and no recurrence for over 2 years. It is important to closely monitor tendon re-rupture and tumor recurrence. If the tendon re-ruptures or the tumor recurs, tendon repair or radical resection of the tumor must be performed [21]. In addition, patients with tendon re-rupture or tumor recurrence need to undergo tendon repair surgery (either tendon suture or tendon grafting based on the degree of tendon defect) [22].

The clinical presentations and radiological findings of patients may initially indicate the presence and margin of a mass and this may be useful for primary diagnosis, surgical planning and postoperative follow-up.However, the final diagnoses must be made on the basis of the histological examination [23].Sometimes,the final diagnoses were inconsistent with the imaging diagnoses,so a biopsy before the surgery should be recommended.The interval between the onset of the first signs and diagnosis is often long, suggesting slow progression of the disease. In this case, even though the symptoms were mild and nonspecific, the possibility of the EIP tendon’s impairment should not be overlooked. Ignoring the clinical presentations and delaying treatment can result in poor prognosis. In conclusion, early diagnosis and decreasing movement of the involved tendon are important to improve the prognosis of patients.

In the case, postoperative pathology showed pervasion of the synovial mononuclear cells and the multinucleated giant cells were dispersed in the interstitial tissue. Immunohistochemical examination also showed the presence of CD68 (+) and Ki-67(+). We found that, in some cases, the cytoplasm of the synovial mononuclear cells contains deposits of iron-hemoflavin particles [24]. Together, these pathological characteristics contributed to making the final diagnosis. Even so,the differential diagnoses of GCTTS from synovitis, hemorrhages, specific and non-specific infectious arthritis, rheumatoid polyarthritis, synovial hemangioma, synovial osteochondromatosis, synovial arborescence lipoma, and synovial sarcoma should be considered [24–26]. Although MRI plays a vital role in initial diagnosis and surgical planning, the final diagnoses are largely made on the basis of histological examination. Additionally, in case of slightest doubt a biopsy should be performed, especially if initial diagnosis confirms the presence of nodular GCTTS.

In conclusion, GCTTS originating from the hands or wrists are usually benign and localized, but in extremely rare cases, there is a possibility of tendon damage. In this report, we detailed a case of GCTTS in the wrist that impaired the extensor indicis proprius tendon. Based on this case, we learned that early radical resection is recommended upon diagnosis,at the same time,the decreasing movement of the involved tendon will be helpful.All these will help to prevent the destructive proliferation of the tumor, maximumly reserve the tendon’s function, and contribute to good prognostic outcomes.

## Data Availability

The dataset supporting the conclusions of this article is included within the article.

## Abbreviations

EIP tendon: Extensor indicis proprius tendon
GCTTS: Giant cell tumor of tendon sheath
MRI: Magnetic resonance imaging
PVNS: Pigmented Villonodular Synovitis
